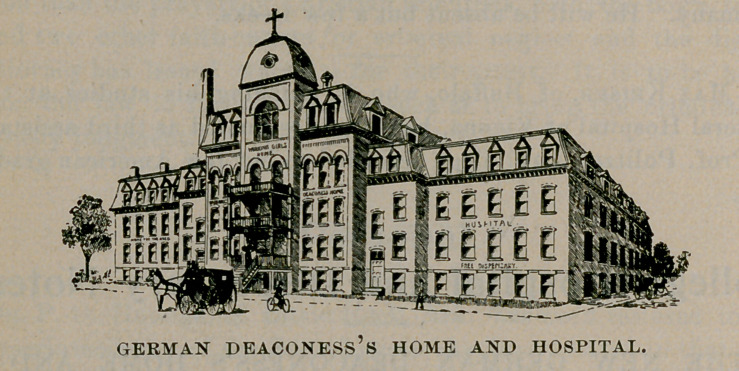# College, Hospital and Dispensary Notes

**Published:** 1897-01

**Authors:** 


### College, Hospital and Dispensary Notes.

### THE NEW GERMAN DEACONESS'S HOME AND HOSPITAL.

ON NOVEMBER 21–26, 1896, occurred the dedication ceremonies of the new German Deaconess's home and hospital, situated on Kingsley street near Humboldt parkway. As the hospital division promises to be a leading feature, and therefore of interest to the profession of Buffalo and vicinity, a short sketch of the building and purposes of the association may be of some interest to the readers of the Journal.

About two years ago, the idea of establishing in Buffalo a Deaconess's Home, Hospital and Home for the Aged, was first conceived in the minds of a few of Buffalo's worthy citizens. A number of private meetings were held, and, after careful deliberation, it was decided to call a public meeting to present the plan to the people. Such a meeting was held on February 26, 1895, in the St. Paul's German U. E. church, on Ellicott street. The interest and enthusiasm manifested was such as justified the organisation of a society whose object is to further the interests of the work. This society is now known as the Deaconess's Association of Buffalo.

On October 23, 1895, the association rented a building on Goodrich street, and the first patient was admitted on November 14, 1895. The success of the venture was positive from the outset, and in the spring of 1896 the erection of a new and commodious building was taken into consideration. Without unnecessary delay a desirable site and appropriate plans were secured, and the process of building was begun. Now, after an elapse of but thirteen months, the association is enabled to open to the public a structure of which it can justly be proud. Though plain and unpretentious in appearance, it is well adapted to its uses, and is a

GERMAN DEACONESS'S HOME AND HOSPITAL.

noble addition to the new and charitable institutions of our beautiful city. The home is constructed in three distinct divisions: the central, or main division, is intended as a home for deaconesses and working women; the east wing will be used for hospital purposes, and the west wing as a home for aged men and women. Each division will accommodate about forty inmates. The east wing, containing the hospital department, is arranged as follows: The basement is divided up into a kindergarten room, which is a sort of creche, intended for use when the work develops, though nothing will be done with it at present; a polyclinic waiting-room, drug department and office. On the first floor are two wards for men, a small children's ward, five private rooms, a day room and a diet kitchen. On the second floor is the operating room, connecting with a medicine and preparing room. There are also two wards, five private rooms, a day room and a diet kitchen for the women patients, and on the third floor are eight rooms to be fitted up in case of necessity.

The medical staff of the hospital comprises the following physicians: Consulting physicians, Conrad Diehl, M. D., Louis Schade, M. D., Charles Wetzel, M. D.; attending physicians, DeLancey Rochester, M. D., E. S. Tobie, M. D., Wm. Gaertner, M. D.; attending surgeons, Herman Mynter, M. D., Roswell Park, M. D., Eugene Smith, M. D.; gynecologist, M. D. Mann, M. D.; ophthalmologist, Edmund Blaauw, M. D.; laryngologist, W. S. Renner, M. D.; dermatologist, Alfred Diehl, M. D.; diseases of children, Irving M. Snow, M. D.; neurologists, James W. Putnam, M. D., Wm. C. Krauss, M. D.; pathologists, Herbert Williams, M. D., Earl Lothrop, M. D.; obstetrician, Henry G. Bentz, M. D.; resident physician, Dr. Graff.

The success of this undertaking is due largely to the persistent and sacrificing efforts of the Rev. Carl Schild, who very properly becomes president of the board of directors, to the generosity of J. F. Schoellkopf, and the medical department owes much to the thoughtful care of Dr. Eugene A. Smith. The management of the home is under the general supervision of the sister superior, Miss Tobschall, known to the inmates of the home as Sister Ida. Miss Mary Barth, a graduate of the Buffalo General Hospital, has been engaged as superintendent in the hospital department of the new building. Mrs. Eliza Loy will have the supervision of the home for the aged.

---

THE German Dispensary, lately organised in Buffalo, was opened December 14, 1896, at 621 East Genesee street, with the following staff: President, Chas. H. W. Auel, M. D.; vice-president, Gustave Pohl, M.D.; secretary, Max Breuer, M.D.; house committee, Drs. L. Schroeter, S. Goldberg, Henry G. Bentz; general practice, Drs. E. E. Koehler, Henry Osthues, Fr. Thoma, Julius Ullman; surgery, Drs. J. G. Meidenbauer, M. Hartwig; consulting surgeon, Dr. Herman Mynter; diseases of women, Drs. Chas. H. W. Auel, Max Breuer, S. Goldberg; psychiatry and diseases of the nerves, Drs. William C. Krauss, H. G. Matzinger, William Meisburger; diseases of children, Drs. L. Schroeter, G. Pohl, C. H. W. Auel; ophthalmology and otology, Dr. E. Blaauw; consultant, Dr. Lucien Howe; genito-urinary and skin diseases, Drs. J. M. Kraus, A. Jokle, G. W. Wende; consultant, Dr. Ernest Wende.

The present number of active members is twenty-two, which may be raised to the limit of twenty-five. There are three consulting physicians and surgeons as above indicated.

The aim of the dispensary will be to accept none but patients absolutely too poor to pay a fee. A committee of three directors will act as investigating committee, scrutinising the list each week with a view to ascertain if any patients not entitled to charity are being treated. It is expected that this committee will communicate with the district physicians of the health department, also every attending physician at the dispensary will make himself familiar with the social condition of each patient, rejecting such as are found able to pay for medical treatment. If the dispensary staff carries out these well-formed resolutions to the letter it will be entitled to the confidence of the community.

## Figures and Tables

**Figure f1:**